# Representation of Black, Asian and minority ethnic patients in secondary care mental health services: analysis of 7-year access to memory services in Leicester and Leicestershire

**DOI:** 10.1192/bjb.2020.3

**Published:** 2020-08

**Authors:** Hari Subramaniam, Elizabeta B. Mukaetova-Ladinska, Andrew Wilson, John Bankart

**Affiliations:** 1 The Evington Centre, Leicestershire Partnership NHS Trust, UK; 2 Department of Neuroscience, Psychology and Behaviour, University of Leicester, UK; 3 Department of Health Sciences, College of Life Sciences, University of Leicester, UK

**Keywords:** BAME service use, memory clinic, ethnicity, referrals

## Abstract

**Aims and method:**

We aimed to explore access by Black, Asian and minority ethnic (BAME) elders to the memory services in Leicester and Leicestershire, examining any trends over time. We then compared the odds of referral by ethnicity, using observed versus expected referrals for the city of Leicester. We gathered data on a comprehensive county-wide memory clinic used by people with suspected dementia and memory problems from the Trust electronic record system during the period 2011–2017. For Leicester city, we compared referral rates for 2011–2017 and compared observed and expected referral rates with demographics from the UK Census 2011.

**Results:**

In Leicester, there was a significant underrepresentation of referrals from the BAME population as compared with the White population in 2011, 2012 and 2013, when compared with population estimates of those aged ≥60 years from the 2011 UK Census Leicester city data. Data for the Black population were too small for comparisons. The odds of being referred to a memory clinic for the White group was double that of the Asian group in 2011 (odds ratio 2.15, 95% CI 1.52–3.02) and nearly 1.5 times in 2012 (odds ratio 1.40, 95% CI 1.01–1.93). This difference did not persist after 2014. However, this differential odds of referral changes when the age difference between the groups is accounted for. After adjusting for age, there were no differences between the two groups in their odds of referral to the memory clinic from 2011 to 2013, but from 2014 to 2017, members of the Asian group had higher odds of being referred.

**Clinical implications:**

The relationship between BAME and access to memory services is complex. The relative lower prevalence of Asian people among referrals to memory services in Leicester from 2011 to 2013 may partly be explained by the lower ages of the Asian population at referral. The higher prevalence of Asian people in 2014–2017 may be owing to use of denominators from the 2011 UK Census, which are likely to be disproportionately low for this group. Further studies are needed to explore any potential barriers to the access of services by BAME communities.

There are an estimated 25 000 people currently living with dementia from Black, Asian and minority ethnic (BAME) backgrounds in the UK.^1^ It is estimated that this figure will rise to 50 000 by 2026 and 172 000 by 2051, given the growing and ageing UK BAME population.^2^ This increase parallels the projected increase in the numbers of older BAME people. In 2001, approximately 532 000 people from BAME groups were aged ≥65 years, and this is expected to rise eightfold to approximately 3.8 million by 2051.^3^

BAME people are generally underrepresented in dementia services and often present later in the course of the illness^4^; development of appropriate health and social care services to meet their needs has been a longstanding priority. A national study^5^ found that the incidence rates of dementia diagnosis are higher among Black ethnic groups compared with White and Asian groups in the UK. This study reported that the incidence of dementia diagnosis was 25% higher among Black women than White women, and 28% higher among Black men than White men. Asian women and men were 18% and 12% less likely than White women and men, respectively, to have a dementia diagnosis. Vascular dementia is thought to be more common among Asian and Black Caribbean people because they are more prone to important risk factors for vascular dementia, such as cardiovascular disease, hypertension and diabetes,^6^ and some evidence suggests that people from BAME groups present later in their illness.^7^

In the UK there are primary care-based studies estimating the prevalence of dementia diagnosis rates^8^ in BAME groups, but there are not many county-wide studies on mental health services use within secondary care services by people with dementia. UK-based secondary care studies tend to have small population sizes; a recent study^9^ examined data on referrals to an inner London memory service to explore any differences in referral rates, cognitive assessments and stages of dementia at presentation. Although Black and Black British patients comprised a quarter of all referrals, Asian patients comprised only about 2.6% of the referrals. Another secondary care study^10^ paradoxically found BAME overrepresentation, but this is the only UK study to find this. Such studies that are small and with seemingly opposite findings suggests that referral patterns may not necessarily reflect the BAME distributions in the local population and could well be representative of other factors. Further, it emphasises the need for studies with larger BAME population samples.

Several qualitative studies have explored why BAME groups may be reluctant to seek professional help for symptoms owing to dementia.^4,11–13^ There are several barriers to seeking help identified for South Asian people. They include patient, carer and community factors, health professional factors and the type of services concerned.^14^ Health professional factors include culture and communication, lack of knowledge of dementia, lack of knowledge of available services (including any for BAME groups) and high workload/lack of time. There are several other reported barriers, such as lack of support, time and financial constraints, stigma, diagnostic uncertainty and disclosure of the diagnosis.^15^ Furthermore, interventions raising awareness^16^ have not shown much improvement in diagnosis or management,^17^ which might suggest an associated reluctance to seek help.

## Demographic characteristics of Leicester and Leicestershire

Leicester is unique in the UK in probably now being the only city to have a sufficiently large BAME population (with only 51% reporting themselves as White British) in the UK Census 2011 data; population changes have been affected over time by immigration patterns. Further, Leicester is also unique in having a South Asian population as the majority within the BAME population. Additionally, Leicester has the highest proportion of BAME adults of any city in the UK, with the BAME population being the majority population in some areas. The Leicester data from the 2011 UK Census gave the three main ethnicity estimates in the city to be 51% White, 37% Asian and 6% Black.^18^ The Leicester Action Plan 2016/7 estimates that 3000 people aged >65 years in Leicester live with dementia, and this is forecast to increase to about 4500 people by 2030.^19^ Estimates suggest that each year about 850 people diagnosed with dementia are from Black and minority ethnic backgrounds.^20^ Leicester has a high-performing diagnosis rate for dementia of 88.4% of the predicted population value.^20^

The demographics of Leicester show that the increase in the proportion of those aged >65 years is much greater in the South Asian population than other groups, and that overall, the proportion of this age group from BAME communities will have risen from 31% in 2016 to 40% in 2026.^19^ This raises the question of whether local referral patterns reflect the real prevalence of the Asian population among BAME referrals. It provides us with a good opportunity to explore secondary care service use by BAME groups in a setting where the BAME population is high, in a city with only about 51% reporting themselves as White British in the UK Census 2011 data.^18^

## Aims

First, we wished to examine the access by BAME elders to memory services in Leicester and Leicestershire and examine any trends over time. Second, we aimed to compare the odds of referral by ethnicity, using observed versus expected referrals for the city of Leicester.

## Method

### Data collection

We gathered data on comprehensive county-wide memory clinic use within mental health services by people with suspected dementia and memory problems. For this we used the Leicestershire Partnership National Health Service Trust (LPT) electronic record system, RiO.

The LPT moved to an electronic system of patient records in 2010 and this included electronic records of all referrals to the memory clinics, out-patient referrals and referrals to the community mental health teams. As a part of a National Institute of Health Research (NIHR) study, the findings of which will be reported elsewhere, we identified all referrals to the LPT memory clinics where patients suspected of cognitive disorders and dementias would be referred and diagnosed. Memory clinics provide assessment for cognitive problems across the city and county to adults referred with suspected memory problems. Patients are offered comprehensive assessments, including standardised tests, brain imaging and neuropsychological tests as needed. Diagnoses are considered by a multidisciplinary team and appropriate treatments and follow-up are arranged as required.

For this study, we report only the findings of ethnicity data and findings related to patients referred to mental health services for the elderly who were referred by general practitioners for memory assessment. Ethnicity was coded as per the National Health Service (NHS) criteria for recording ethnicity data at the time of referrals. We were able to stratify the data according to the ethnicities as recorded on NHS patient-recording systems. The data collected included data for older people referred for a memory assessment, but not those suspected of having a young-onset dementia or cognitive syndromes in younger adults, this information being reported separately. We collected date of referral, ethnicity recorded at the point of receiving the referral, gender, general practitioner details and whether the case was active or discharged. Age was not routinely recorded at the time of receiving the referrals.

We collected data for 8 years, from 1 January 2010 to 31 December 2017. Analysis used anonymised data from a yearly referral database with LPT maintained for the purpose of service monitoring and includes all the referrals received by the Trust. NHS ethnic data categories include White British, White Irish, Asian or Asian British, Black or Black British, any other White, mixed, Chinese, other ethnic, not known and not stated. We studied patterns yearly to look for any emerging trends.

Ethics approval for this study was granted by University of Leicester as a part of the NIHR study application (reference PB-PG-0416-20019). The study also had approval from the LPT Research and Development Department (ELMH0818; Integrated Research Application System reference 232861).

### Age imputation

The age distribution of the entire Leicester city population at risk was already known for 2011 in 5-year age bands. As age was not routinely recorded at the time of receiving referrals, we collected these data only for a sample of randomised individuals. Because we lacked age information for the non-randomised referrals, we decided that it was reasonable to assume that the age distribution for the randomised referrals (in 5-year bands) would be the same as for the non-randomised referrals, and imputed the age data on that basis. So, if a third of randomised White referrals in 2011 had ages in the 80–84 years age band, the same proportion was imputed for non-randomised White referrals in 2011. This resulted in the creation of seven data-sets, one for each year (2011–2017). Age distributions were calculated and created separately for the non-randomised referrals in the two ethnic groups in each year. In each year, the total number at risk was the same, so no account was taken of population growth in those aged ≥60 years, for which we had no information. The total number at risk in each year consisted of 49 115, with the number at risk being constrained to be constant from 2011 to 2017. The number of referrals in each year was subtracted from the number at risk to give the number of non-referrals. The numbers of non-referrals in the years subsequent to 2011 were not removed from the at-risk group to take into account the fact that the referrals in previous years may no longer have been at risk of being a referral. Having estimated the numbers of referrals and non-referrals in each age band in each year for each ethnic group, we generated these data in Stata version 14 for Windows, using the ‘gen’ command.

### Statistical analysis

A separate analysis was performed for each year, and *P*-values were correspondingly adjusted for multiple comparisons with the Bonferroni method (significance level 0.05/*n*, two-tailed). Logistic regression was used to compare proportions of referrals between ethnic groups, using 5-year age bands to calculate age-adjusted odds ratios and 95% confidence intervals. Population-at-risk estimates were derived from the published UK Census (2011) data for Leicester. Age was only available in the form of six (mainly 5-year) age bands, ranging from 60–64 to ≥85 years and was treated in the logistic models as a continuous predictor, ranging from 1 (60–64 years) to 6 (≥85 years). We used 60 years as the cut-off for population-at-risk estimates, as it is reflective of the age generally used as a cut-off age for population-at-risk estimates in defining older adult services and has face validity in clinical practice. However, beyond 2013 it is highly likely that the denominators for the two ethnic groups will have both increased and also diverged non-trivially, leading to potential biases in the estimated proportions and differences in proportions, so estimates of individual proportions and differences in proportions beyond this time should be treated with caution.

### Randomisation

Simple randomisation by a computerised program (SAS version 9.4 for Windows) had previously been carried out from the memory clinic referrals in the White and Asian groups separately, to obtain a representative sample of 260 cases from each group; the ages of referral were then individually collected from those case notes for comparison. The number of Black referrals was too small for meaningful comparisons, and hence this study did not include data for the Black population. All analyses were exploratory.

## Results

Over the analysed period, LPT memory services received a total of 15 634 referrals, of which 191 (1.2%) had been entered in error. These included referrals that were deemed inappropriate or had other medical problems present and hence were not suitable for memory clinic assessments. A total of 1493 (9.6%) people had blank values in the ethnicity data rows, whereas ‘not known’ ethnicity was recorded in 994 (6.4%) people. These missing data were missing at random with no consistent pattern, and were all excluded from the analyses. Formal statistical analyses were conducted on just the two main groups of interest here, namely White British and Asian groups; other ethnic groups and mixed groups were excluded because of the low numbers in each group. Comparisons of the main groups are shown in Table 1.

**Table 1:** Referral data for Leicestershire memory clinics, 2011–2017

Referrals by ethnicity	*N*	Gender		% Female	Percentage of the sample, *N* = 15 443
		Male	Female		
White British	11 166	4631	6535	58.5	72.3
White Irish	152	65	87	57.2	1.0
Asian or Asian British	1128	476	652	57.8	7.3
Black or Black British	141	67	74	52.4	0.9
Any other White	288	93	195	67.7	1.9
Mixed	35	17	18	51.4	0.2
Chinese	9	3	6	66.6	0.06
Other ethnic	37	16	21	56.7	0.2
Not known	994	453	540	54.3	6.4
Not stated	1493	651	842	56.3	9.7

Despite the overall higher proportion of BAME in Leicestershire compared with many other counties, during the period 2011–2017, of all the referrals across the county, only 1128 were Asian (7.3%) and 142 were Black (0.9%).

The proportion of Asian ethnicity referrals was higher in the city of Leicester (Table 2), but it was still only 22.6% compared with the expected Asian proportion of 37% based on the 2011 UK Census data population size estimates.

**Table 2:** All memory services referrals for Leicester city and county, 2011–2017

Total referrals	Referrals	White	White, %	Asian	Asian, %	Black	Black, %
City	4182	2529	60.5%	946	22.6%	120	2.9%
County	11 452	8637	75.4%	182	1.6%	22	0.1%
Total	15 634	11 166	71.4%	1128	7.2%	142	0.9%

### Yearly comparison of referrals in Leicester city and Leicestershire county

We have presented the referral rates among the three largest ethnic groups over the period 2011–2017 (see Table 3). Referral numbers increased consistently in all the groups for part of this period. There were 529 referrals combined in all the three groups in 2011, peaking at 3313 in 2016, but then decreasing to 2033 in 2017. This drop may be explained by the increase recorded in the ‘not known’ and ‘not stated’ ethnic categories. White British referrals increased from 461 in 2011, peaking at 2350 in 2015 and dropping to 1337 in 2017. Asian ethnicity referrals also rose from 43 in 2011, peaking at 295 in 2015. Black ethnicity referrals were relatively low throughout, ranging from 5 in 2011 to 25 in 2017.

**Table 3:** Annual referrals for Leicester and Leicestershire (*n*, % referrals)

	White British	Asian	Black	Other White	Not known	Not stated	Total
	*n*, %	*n,* %	*n*, %	*n,* %	*n*, %	*n,* %	
2011	461, 87.1	43, 8.1	5, 0.0	9, 1.7	2, 0.3	2, 0.3	529
2012	870, 87.5	43, 4.3	4, 0.0	20, 2.0	17, 1.7	7, 0.7	994
2013	1422, 76.0	113, 6.0	18, 0.9	44, 2.3	195, 10.4	45, 2.4	1870
2014	2142, 76.5	228, 8.1	28, 1.0	53, 1.8	254, 9.0	51, 1.8	2800
2015	2350, 78.5	294, 9.8	25, 0.8	64, 2.1	134, 4.4	201, 6.7	2991
2016	2068, 62.4	242, 7.3	25, 0.7	55, 1.6	166, 5.0	725, 21.8	3313
2017	1338, 65.8	148, 7.2	26, 1.2	28, 1.3	226, 11.1	458, 22.5	2033

The proportion of White British referrals fell over this period from 86.9% in 2011, to 65.6% in 2016 and 65.7% in 2017 (Table 3). In contrast, referrals from the Asian population remained relatively similar over this period, from 8.1% in 2011, peaking at 9.8% in 2015 and then slightly falling again to 7.2% in 2017. The Black population proportion remained low, rising from 0.09% in 2011 to 1.2% in 2017.

### Comparison with Leicester city population estimates

As the raw referral rate data suggested a lower referral rate among BAME groups compared with the White British population, for Leicester city we compared annual referral rates between 2011 and 2017, based on an estimate of the population at risk, derived from the 2011 UK Census. We obtained this population-at-risk estimate by an age-defined cut-off of 60 years, obtained by stratification of the known population estimates taken from the 2011 UK Census data. A cut-off age of 60 years holds greater relevance and reflects the age cut-offs normally associated with the way services reflect clinical practice. We restricted this analysis to Leicester city as the city has a sufficiently large BAME population to make statistical comparisons meaningful. It is interesting to note that the referral patterns from the city rose from 2011, peaking in 2016, but fell again in 2017 (Table 4).

**Table 4:** Comparison between Asian and White groups in Leicester city (unadjusted)

2011 UK Census	White		Asian		Comparison
	166 636		122 470		
Population at risk (*n* > 60)	34 750		14 365		
	*n*	Rate per 1000	*n*	Rate per 1000	White (reference) versus Asian, odds ratio (95% CI)
2011	207	5.96	40	2.78	2.15 (1.52–3.02)
2012	162	4.66	48	3.34	1.40 (1.01–1.93)
2013	357	10.27	100	6.96	1.48 (1.18–1.85)
2014	485	13.96	191	13.30	1.05 (0.88–1.25)
2015	526	15.14	253	17.61	0.86 (0.73–0.99)
2016	452	13.01	188	13.09	0.99 (0.83–1.18)
2017	265	7.63	123	8.56	0.89 (0.71–1.11)
Total	2454	7.06	943	6.56	1.08 (1.00–1.17)

To control for the effects of age at presentation, we compared the White ethnicity and Asian ethnicity groups, using age-adjusted logistic regression over the period 2011–2017 (Table 5), with data from each year being analysed separately. There is a clear trend between 2011 and 2015 showing the odds ratio changing increasingly in favour of Asian patients being referred.

**Table 5:** Comparison of Asian and White groups in Leicester city in 2011–2015, adjusted for age (results 2011–2017)

Year	Odds ratio (95% CI) for ethnicity 1/odds ratio	*P*-value for ethnicity	Odds ratio (95% CI) for age	*P-*value for age	Pseudo *r*^2^
2011	1.13 (0.80–1.60) 0.93	0.48	2.92 (2.57–3.32)	<0.001	0.170
2012	0.87 (0.62–1.22) 1.15	0.43	2.03 (1.83–2.24)	<0.001	0.096
2013	0.80 (0.63–1.03) 1.25	0.06	2.68 (2.46–2.92)	<0.001	0.171
2014	0.72 (0.60–0.86) 1.37	<0.001	1.74 (1.65–1.83)	<0.001	0.077
2015	0.51 (0.43–0.60) 1.92	<0.001	2.19 (2.07–2.31)	<0.001	0.138
2016	0.67 (0.56–0.80) 1.49	<0.001	1.80 (1.71–1.90)	<0.001	0.085
2017	0.61 (0.48–0.76) 1.64	0.001	1.77 (1.65–1.89)	<0.001	0.074

Reference category for ethnicity is Asian (coded as 0). Age is modelled as a linear and continuous variable, so for 2011, for every rise in age category (5-year bands), the odds of being referred increase by a factor of nearly 3. After Bonferroni correction for multiple comparisons (0.05/14), the adjusted significance level becomes 0.003, so all results for 2014–2017 are significant.

At any given time only a proportion of patients referred for a memory clinic were actively being managed within the service. Some would be waiting for an assessment and some would have been assessed, treated and discharged. To get a fair representation, we compared the numbers of referrals that were considered actively open to see if they matched estimates of patients with suspected dementia in Leicester. As of 2017, there were 932 open cases in the city, with White British cases being 54% of the total. We compared the active cases from the three groups with their at-risk estimates in Leicester city (based on the Leicester 2011 UK Census). Statistical comparison of active memory clinic use data shows significantly lower use by BAME groups. The odds of being actively open to the memory clinic were 1.67 (95% CI 1.42–1.96; *P* < 0.0001) times lower in the Asian population (24% of active cases compared with the 40% of total at-risk Asian population estimates), whereas the odds of being actively open to the memory clinic were 2.72 (95% CI 1.79–4.15; *P* < 0.0001) times lower for the Black population (among the 2.4% of active cases compared with the 7% of total at-risk Black population estimates).

## Discussion

Referrals of patients to memory services in Leicester and Leicestershire have increased fourfold over the period 2011–2017, although the drop of 39% between 2016 and 2017 is not easy to explain. We found that Asian people represented 22.6% of all the memory service referrals in Leicester city and 1.5% within the county of Leicestershire. The Black population appears to be severely underrepresented among referrals to the service.

Referrals from White British groups rose sharply from 2011 to 2014, but then stabilised. Interestingly, the referrals from the BAME groups have correspondingly not increased, suggesting the role of other factors (i.e. access difficulties, immigration changes) that need to be accounted for. However, this could be partly explained by the higher proportion of ‘not known’ or ‘not stated’ ethnicity groups. The role and the nature of the assessments in memory clinics have also perhaps changed over these times, with increasing awareness of the newer concept of minimal cognitive impairment and changes to the assessments of cognitive issues associated with functional illness and/or physical illnesses. There could also be influences arising out of the National Dementia Strategies^21^ and the changes within primary care (such as Quality Outcomes Framework targets)^22^ or the changes in costs associated with anti-dementia drug prescribing. This may mean that the population presenting to memory clinics for assessment may have altered in its composition over the years, with a greater emphasis on early assessment for cognitive problems. Administrative reasons may affect data collection, explaining the higher ‘not stated’ scores, and perhaps political influences affect the ethnicity documentation or the ‘not known’ scores. We suspect these uncoded data may also affect the ongoing activity and open case contacts, and may need to be taken into account when interpreting the results.

In this study we demonstrate underrepresentation of Asian ethnicity groups in Leicester city memory clinic referrals in 2011, 2012 and 2013 when we compare them with unadjusted population-at-risk estimates derived from the Leicester BAME demographic data from the 2011 UK Census. However, this difference can be explained by the finding that the Asian population is younger than the White population at the time of the referral. After adjusting for age, there were no ethnic differences between the two groups in their odds of being referred to memory clinic before 2014, from which time the denominators become increasingly unreliable. Age is thus the more important predictor of being referred to memory services. For every rise in age category (5-year bands), the odds of being referred increased by a factor of around 1.5 to 3. There is a clear trend between 2011 and 2015 showing the odds ratio changing in favour of Asian people being referred. There may be two main reasons for this. First, this is likely to be because of the denominator for the Asian population increasing more than the denominator for the White population, leading to increasingly high numbers at risk for Asian people relative to White people. However, we could not take this into account in the analyses as the data which could confirm this are not available. Second, it is also possible that the clinical presentations in this group may be such that general practitioners feel more inclined to refer to memory clinic for a specialist assessment. We cannot identify any other factors that might change the likelihood of Asian people being referred compared with White people, regardless of the number at risk, and there are no changes that we can identify in referral methods or local clinical practices.

As far as we know, this is the first comprehensive study of BAME referral rates at a county-wide level within secondary care services. Although there have been other studies looking at secondary care memory clinic use, they have been confined to district or borough levels, often covering a few memory clinics and community mental health teams. This study's strength is that it covers the whole of Leicester/Leicestershire, which has multiple memory clinics and covers all the community mental health teams in the county. By that nature, our study is comprehensive and cover practices across an entire healthcare system.

Reinforcing the findings from other UK studies, our findings also suggest underuse of services by BAME groups within secondary care memory services; however, the lower odds in the BAME group of being referred to services may be explained by their lower ages at the time of referral. The odds of getting referred to memory services are changing, with the odds ratio favouring Asian people being referred in the latter years of the sequence. However, this finding is likely to be owing to underestimation of the population at risk for this group. This is an important finding as Leicester has a very high BAME (chiefly Asian) population in inner city areas and so arguably has sufficient BAME populations to study trends in service use by BAME (chiefly Asian) groups. A study such as ours helps in adding substantively to findings in this area, where there have previously been contradictory reports.

Our findings reinforce the need for more in-depth research to identify reasons for varying presentation of BAME patients in memory clinics and mental health services across different regions and also across different generations.

### Limitations

Despite the comprehensiveness of the study, the numbers in the BAME population in Leicester are relatively small. It is possible that with greater numbers and larger studies across regions, the outcome may be different. Moreover, Leicester's geographical and historical immigration patterns are unique and a similar study elsewhere may have different findings. Consideration should also be given to the role of the primary care physicians and the diversity of the ethnic backgrounds they may come from, which could affect referral practices. A major limitation is using the age data from the 2011 UK Census to adjust rates beyond the year of the census. The population profile would have changed since the 2011 UK Census data estimates, and comparing the referral rates in the latter years with this data would limit its applicability, but the 2011 UK Census data remains the last officially published national estimates of UK population data. Also, an at-risk population with an alternate cut-off age other than 60 years may result in different findings.

There may be other reasons apart from age and ethnicity that could also explain the underrepresentation of BAME patients in our sample. Additional missing variables relate to physical morbidity and health service use elsewhere (e.g. acute physical health services), traditional cultural practices and reluctance in seeking help from Western services, the role of the extended family system, and the perception of the inevitability of dementia and it being seen as a part of normal aging decay. The barriers these pose should be explored in further studies.

This study is limited by the way ethnicity is coded by NHS staff at the point a referral is received. Further, the categories have been broadly classified; not analysing further subtypes of ethnicity and its clinical implications may be a limitation, but it was beyond the scope of this study. Similarly, there are changing migration patterns and intergenerational differences, which again are beyond the scope of this study.

In light of these limitations, caution is needed in interpreting the findings. BAME groups by their nature are heterogeneous and subject to constant change, owing to cultural, immigration or political influences. BAME groups may vary in different geographical regions and may be affected by other factors, such as economic indicators and deprivation. It is possible that the BAME groups in Leicester may be economically not as deprived as in other areas such as the north of England, and the pattern of referrals to memory services in such areas may be different. Furthermore, there are intergenerational effects and as such a repetition of this study in the coming decades may reveal different findings.

### Future work

Future work is needed to carry out additional investigations into any perceived barriers to help-seeking in BAME populations. We are currently in the process of undertaking an NIHR-funded study to look at diagnostic challenges and the severity of presentation of dementia in BAME populations, and this will be reported in due course.

## About the authors

**Hari Subramaniam** is a Consultant Psychiatrist at The Evington Centre, Leicestershire Partnership NHS Trust, and Honorary Senior Lecturer at the University of Leicester, UK. **Elizabeta B. Mukaetova-Ladinska** is a Consultant Psychiatrist at The Evington Centre, Leicestershire Partnership NHS Trust, and Professor of Old Age Psychiatry in the Department of Neuroscience, Psychology and Behaviour, University of Leicester, UK. **Andrew Wilson** is a Professor of Primary Care Research in the Department of Health Sciences, College of Life Sciences, University of Leicester, UK. **John Bankart** is a Honorary Associate Professor in Medical Statistics in the Department of Health Sciences, College of Life Sciences, University of Leicester, UK.
